# Serotonin Transporter (*5-HTT*) Gene Polymorphisms and Susceptibility to Chronic Periodontitis: A Case-Control Study

**DOI:** 10.3389/fgene.2019.00706

**Published:** 2019-08-05

**Authors:** Lan Wu, Tong Deng, Chao-Yang Wang, Xue-Qun Ren, Yun-Yun Wang, Xian-Tao Zeng, Pei-Liang Geng

**Affiliations:** ^1^Center for Evidence-Based and Translational Medicine, Zhongnan Hospital of Wuhan University, Wuhan, China; ^2^Department of Stomatology, Zhongnan Hospital of Wuhan University, Wuhan, China; ^3^Center for Evidence-Based Medicine, Institute of Evidence-Based Medicine and Knowledge Translation, Henan University, Kaifeng, China

**Keywords:** *5-HTT*, polymorphism, chronic periodontitis, PCR-RFLP, case-control study

## Abstract

**Objective:** The current study is aimed at exploring the relationship between chronic periodontitis and serotonin transporter (*5-HTT*) gene polymorphisms (rs6354 and rs12449783) in the Chinese Han population.

**Methods:** This study included a total of 120 patients with chronic periodontitis and 125 healthy control subjects. The *5-HTT* gene (rs6354 and rs12449783) was genotyped using oral mucosal tissue with a polymerase chain reaction-restriction fragment length polymorphism (PCR-RFLP). Linkage disequilibrium was examined using Haploview. Genotype and allele frequencies were compared between the cases and controls using a χ^2^ test.

**Results:** Genotype distribution of the *5-HTT* gene polymorphisms rs6354 and rs12449783 in the control group conformed to Hardy–Weinberg equilibrium. The frequency of the AC genotype, the AC + CC genotype and C allele of the *5-HTT* rs6354 polymorphism was higher in cases (*P* < 0.05) vs. the healthy control. The adjusted odds ratio (OR) was 1.910 (95%CI = 1.049–3.476) for the AC genotype, 2.026 (95%CI = 1.115–3.680) for the AC+CC genotype, and 1.875 for the C allele (95%CI = 1.089–3.228. Such an association was particularly strong in women for the AC genotype (OR = 2.167, 95%CI = 1.034–4.542). The genotype and allele frequencies of rs12449783 did not differ between the cases and controls. Haplotype C-C (rs6354-rs12449783) was also more frequent in the cases (OR = 2.372, 95%CI = 1.154–4.875, P = 0.016).

**Conclusion:** Chronic periodontitis is associated with the *5-HTT* gene rs6354 polymorphism, as well as rs6354/rs12449783 interaction.

## Introduction

Periodontal diseases are a set of highly prevalent inflammatory diseases that affect the tissues (gingiva, periodontal ligaments and alveolar bone) that support the teeth (Carinci et al., 2013). In the early stage of gingivitis, only the gums are affected. In the late stage of chronic periodontitis, bone loss occurs. Genetic, environmental, and behavioral factors contribute to the development of periodontal diseases (Meyle and Chapple, 2015; Lee et al., 2015; Silva et al., 2015; Thyvalikakath et al., 2015; Fann et al., 2016). Periodontal diseases could produce a reciprocal impact on general health and other organ systems, and may contribute to the development and progression of diabetes, cardiovascular diseases, gastrointestinal tract diseases, and kidney diseases (Bansal et al., 2013; Nagpal et al., 2015; Carrizales-Sepulveda et al., 2018).

5-hydroxytryptamine (5-HT) is a monoamine neurotransmitter that regulates the function of the central nervous system (Ma et al., 2015) as well as other physiological processes (Chilmonczyk et al., 2015). Re-uptake by the 5-HT transporter (5-HTT or SLC6A4) is a major mechanism that removes 5-HT from the synaptic cleft after release. The human gene is located on chromosome 17q11.2 and consists of 15 exons. The gene occurs in polymorphic forms, which in turn affects 5-HTT expression levels, and ultimately 5-HT concentration (Ho et al., 2013).

The *5-HTT* gene is implicated in complex behavioral traits and a variety of psychiatric diseases (Defrancesco et al., 2013; Cengiz et al., 2015; Tollenaar et al., 2017). Several previous studies associate periodontal diseases with psychological stress and anxiety (Goyal et al., 2013; Graetz et al., 2013; Reshma et al., 2013; Delgado-Angulo et al., 2015). Periodontal disease occurrence is affected by both genetic and environmental factors (Slayton, 2006; Jin et al., 2011; Divaris et al., 2013). 5-HT is also implicated in bone metabolism (Feuer et al., 2015), and could control periodontitis-induced alveolar bone loss (Galli et al., 2013). We therefore speculate that 5-HT neurotransmission is implicated in periodontal diseases and thus conducted a case-control study to examine the potential association between chronic periodontitis with single nucleotide polymorphisms (SNPs) (rs6354 and rs12449783) of the *5-HTT* gene.

## Materials and Methods

### Study Subjects

This study was approved by the Ethics Committee of the Institute of Evidence-Based Medicine and Knowledge Translation, Henan University. Sample collection conformed to the ethics criteria of national human genome research. All subjects provided written informed consent prior to study commencement.

This study included a total of 120 patients with chronic periodontitis (68 women and 52 men; age range: 36 to 57 years) receiving medical care as outpatients at Henan University Hospital during a period from July 2013 to July 2015. Subjects with diabetes or cardiovascular diseases were excluded. The diagnosis was established by the same dentist using the 1999 International Classification of the Periodontal Disease and Conditions (Armitage, 1999). The controls included 125 healthy individuals (72 women and 53 men, aged range: 33 to 64 years) receiving an annual physical check-up at Henan University Hospital during the same period. Controls had no oral diseases, systemic diseases or history of periodontal disease treatment. One-hundred-and-twenty-five healthy individuals (72 women and 53 men, aged between 33 and 64) were included. Both patients and the control subjects received a complete intra-oral examination to assess supra-gingival plaque accumulation, gingival recession, bleeding on probing (BOP), probing pocket depth (PPD), and clinical attachment loss (CAL). Specific criteria for chronic periodontitis included: PPD >5 mm, CAL >4 mm, gingival recession and tooth mobility. Control subjects had no gingival recession, CAL or PPD >3 mm.

### Sample Collection

Oral mucosa was obtained using a sterile dentiscalprum and stored at −20°C prior to genomic DNA extraction using a phenol-chloroform method.

### Genotyping

Primers were designed with Primer Premier 5.0, and synthesized by Sangon Biotech (Shanghai, China) (**Table 1**). PCR amplification was performed in a total volume of 25 µl with an ice bath, including 5 µl 10 × Buffer, 2 µl template DNA, 1 µl upstream primer, 1 µl downstream primer, 0.5 µl Taq DNA polymerase, 2 µl dNTP, and 13.5 µl deionized sterile water. The quality of the PCR products was examined using 1% agarose gel electrophoresis.

**Table 1:** Primer sequences for *5-HTT* gene rs6354 and rs12449783 polymorphisms.

SNP	Primer sequence	
rs6354	Upstream	5′-CCTGCACACTCTTCTCCCTA-3′
	Downstream	5′-TTTCTGCGTTCCCATTATGC-3′
rs12449783	Upstream	5′-AGTAAGCGGTGGCTCACTCC-3′
	Downstream	5′-TGCCCATGTGCATGTTTAAT-3′

PCR products were digested at 37°C overnight with *HpaII* (rs6354) and *MluCI* (rs12449783), respectively. A total volume of 10 µl digestion system contained 2 µl 10 × Buffer, 0.2 µl 100 × BSA, 0.5 µl enzyme, 5 µl PCR products, and 2.3 µl deionized sterile water. DNA fragments were separated using 2% agarose gel electrophoresis and visualized under UV light.

### Statistical Analysis

All statistical analyses were conducted using PASW statistics 18.0 software. Conformity of genotype distribution to Hardy–Weinberg equilibrium (HWE) was examined in the controls with Haploview. A χ^2^ test was used to compare genotype and allele distribution between the cases and controls. Association of *5-HTT* gene polymorphisms with chronic periodontitis is presented as odds ratios (ORs) and 95% confidence intervals (CIs), and adjusted for age, sex and smoking status. A subgroup analysis was performed based on sex. Statistical significance was set at P < 0.05 (two-sided).

## Results

### Demographic and Clinical Characteristics

The obtained 120 patients included 68 women and 52 men with an age range from 36 to 57. And the mean age of the patients and controls was 45.32 ± 10.59 and 46.51 ± 11.86 years, respectively (**Table 2**). The female to male ratio was 1.31 and 1.36 in the cases and controls, respectively. More subjects were smokers in the cases than in controls (*P* = 0.001).

**Table 2:** Demographic and clinical characteristics of the cases and controls.

	Case (*n* = 120)	Control (*n* = 125)	*P*
Age (year)	45.32 ± 10.59	46.51 ± 11.86	0.463
Gender (%)			0.883
Male	52 (43.33)	53 (42.40)	
Female	68 (56.67)	72 (57.60)	
Smoking (%)			0.001
Current and ever	41 (34.17)	19 (15.20)	
Never	79 (65.83)	106 (84.80)	
BOP (% site)	83.55 ± 19.64	47.96 ± 7.1	<0.001
PPD (mm)	5.86 ± 0.67	1.59 ± 0.48	<0.001
CAL (mm)	6.32 ± 0.81	0.0	<0.001

BOP, bleeding on probing; PPD, probing pocket depth; CAL, clinical attachment loss.

### HWE Test

Genotype distribution of the gene polymorphisms rs6354 and rs12449783 in the control group conformed to HWE (*P* > 0.05, **Table 3**).

**Table 3:** Genotype and allele distributions of rs6354 and rs12449783 polymorphisms in the cases and controls.

	Case *n* = 120 (%)	Control *n* = 125 (%)	*P*_HWE_	*χ* ^2^	*P*	OR (95% CI)	*P* *^a^*	**OR^a^ (95% CI)**
rs6354			0.194					
AA	81(67.50)	99(79.20)		−	−	1		
AC	38(31.67)	26(20.80)		3.905	0.048	1.786 (1.001–3.187)	0.034	1.910 (1.049–3.476)
CC	1(0.83)	0(0.00)		1.214	0.271	0.988 (0.964–1.012)	−	−
AC+CC	39	26		4.300	0.038	1.833 (1.030–3.263)	0.021	2.026 (1.115–3.680)
A	200(83.33)	224(89.60)		−	−	1		
C	40(16.67)	26(10.40)		4.126	0.042	1.723 (1.015–2.925)	0.023	1.875 (1.089–3.228)
rs12449783			0.799					
AA	10(8.33)	7(5.60)		−	−	1		
AC	36(30.00)	43(34.40)		0.985	0.321	0.586 (0.203–1.696)	0.446	0.652 (0.217–1.958)
CC	74(61.67)	75(60.00)		0.512	0.474	0.691 (0.250–1.911)	0.714	0.821 (0.285–2.362)
AC+CC	110	118		0.708	0.400	0.653 (0.240–1.774)	0.597	0.756 (0.268–2.132)
CC vs. AA+AC	46	50		0.071	0.789	1.072 (0.642–1.792)	0.564	1.170 (0.687–1.991)
A	56(23.33)	57(22.80)		−	−	1		
C	184(76.67)	193(77.20)		0.020	0.889	0.970 (0.637–1.478)	0.803	1.057 (0.684–1.633)

HWE, Hardy–Weinberg equilibrium; OR, odds ratio; CI, confidence interval.

a the values of P and OR were adjusted by age, gender and smoking.

### Genotype and Allele Distributions

**Figure 1:**
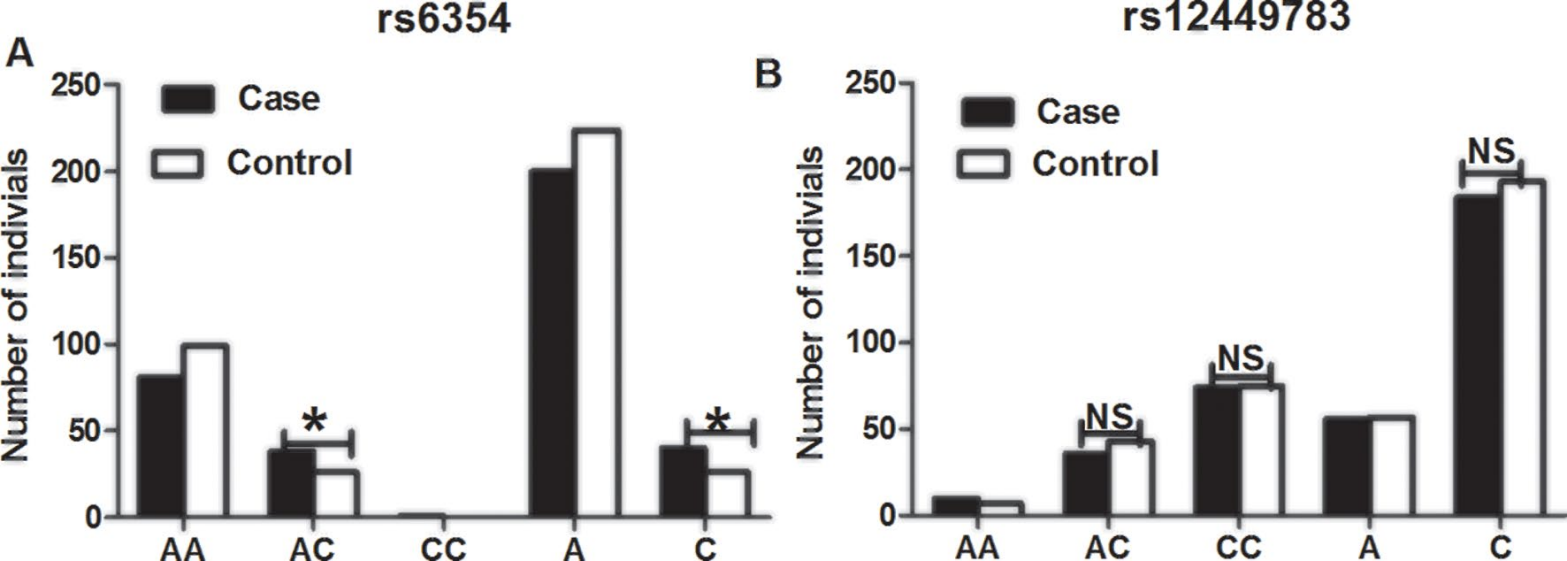
The distributions of *5-HTT* gene polymorphisms rs6354 **(A)** and rs12449783 **(B)** in patients with chronic periodontitis and healthy control subjects. **P < 0.05, NS, not significant.

The CC genotype of rs6354 was not detected in the healthy controls, and in only 1 (0.83%) of the cases (**Table 3** **and** **Figure 1**). The frequency of the AA genotype of rs6354 did not differ between the cases and controls (67.50% vs. 79.20%). The frequency AC genotype of rs6354 was significantly higher in the cases (31.67% vs. 20.80% in the controls; *P* = 0.048). OR was 1.786 (95%CI = 1.001–3.187). An analysis using the dominant model revealed a higher AC+CC genotype frequency in the cases (OR = 1.833, 95%CI = 1.030–3.263, *P* = 0.038). After adjustment for age, sex and smoking, the association remained significant (AC vs. AA: adjusted OR = 1.910, 95%CI = 1.049–3.476, *P* = 0.034; AC+CC: adjusted OR = 2.026, 95%CI = 1.115–3.680, *P* = 0.016). The C allele frequency was higher in the cases (16.67% vs. 10.40%; P = 0.042), with an OR at 1.723 (95%CI = 1.015–2.925). The results were similar after adjustment for clinical parameters (adjusted OR = 1.875, 95%CI = 1.089–3.228, *P* = 0.023).

The rs12449783 genotype did not differ between the cases and controls (**Table 3**): 8.33% vs. 5.60% for AA, 30.00% vs. 34.40% for AC, and 61.67% vs. 60.00% for CC.

**Figure 2:**
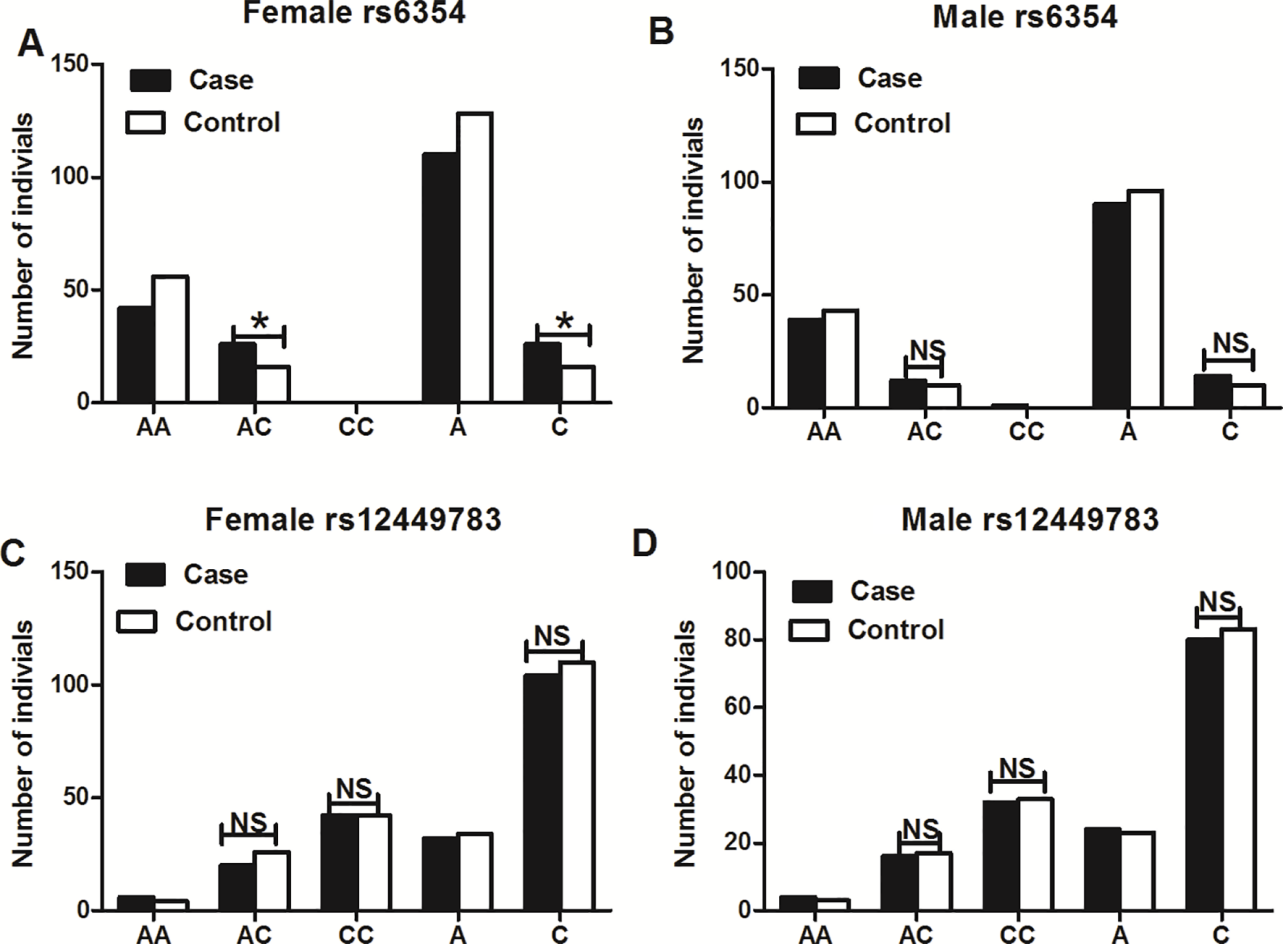
Subgroup analysis of genotype and allele distributions of *5-HTT* gene polymorphisms rs6354 **(A, B)** and rs12449783 **(C, D)**. **P < 0.05, NS, not significant.

The subgroup analysis that included only women showed a higher frequency of the AC genotype of rs6354 in the cases (38.24% vs. 22.22%, *P* = 0.039), with an OR at 2.167 (95%CI = 1.034–4.542) (**Figure 2** and **Table 4**). No difference was detected between the cases and the controls in the male population in both rs6354 and rs12449783 (**Figure 2** and **Table 4**).

**Table 4:** Genotype and allele distributions of rs6354 and rs12449783 polymorphisms: subgroup analysis based on sex.

	Case *n* = 120	Control *n = 125*	*χ*²	*P*	OR(95% CI)
**rs6354**					
Females	*n* = 68 (%)	*n* = 72 (%)			
AA	42 (61.76)	56 (77.78)	–	–	1.000
AC	26 (38.24)	16 (22.22)	4.270	0.039	2.167 (1.034–4.542)
CC	0 (0.00)	0 (0.00)	–	–	–
A	110 (80.88)	128 (88.89)	–	–	1.000
C	26 (19.12)	16 (11.11)	3.517	0.061	1.891 (0.965–3.706)
Males	*n* = 52 (%)	*n* = 53 (%)			
AA	39 (75.00)	43 (81.13)	-	-	1.000
AC	12 (23.08)	10 (18.87)	0.339	0.561	1.323 (0.515–3.402)
CC	1 (1.92)	0 (0.00)	1.088	0.297	1.026 (0.976–1.078)
A	90 (86.54)	96 (90.57)	–	–	1.000
C	14 (13.46)	10 (9.43)	0.841	0.359	1.493 (0.631–3.531)
**rs12449783**					
Females	*n* = 68 (%)	*n* = 72 (%)			
AA	6 (8.82)	4 (5.56)	–	–	1.000
AC	20 (29.41)	26 (36.11)	0.901	0.342	0.513 (0.127–2.065)
CC	42 (61.76)	42 (58.33)	0.358	0.550	0.667 (0.175–2.535)
A	32 (23.53)	34 (23.61)	-	-	1.000
C	104 (76.47)	110 (76.39)	0.000	0.987	1.005 (0.578–1.745)
Males	*n* = 52 (%)	*n* = 53 (%)			
AA	4 (7.69)	3 (5.66)	–	–	1.000
AC	16 (30.77)	17 (32.08)	0.173	0.677	0.706 (0.136–3.658)
CC	32 (61.54)	33 (62.26)	0.158	0.691	0.727 (0.151–3.510)
A	24 (23.08)	23 (21.70)	–	–	1.000
C	80 (76.92)	83 (78.30)	0.057	0.811	0.924 (0.483–1.768)

### The LD Analysis of rs6354 and rs12449783

Significant LD of rs6354 and rs12449783 was identified (D’ = 0.71, r2 = 0.331). The C-C haplotype was associated with periodontitis (OR = 2.372, 95%CI = 1.154–4.875, *P* = 0.016). Detailed haplotype information is shown in **Table 5**.

**Table 5:** Haplotype analysis of rs6354 and rs12449783 polymorphisms.

Rs6354-rs12449783	Haplotype (%)		*χ*²	*P*	OR (95%CI)
	Case	Control			
A-C	159 (66.25)	181 (72.40)	–	–	Ref.
C-A	15 (6.25)	14 (5.60)	0.264	0.608	1.220 (0.571–2.605)
A-A	41 (17.08)	43 (17.20)	0.113	0.737	1.085 (0.673–1.750)
C-C	25 (10.42)	12 (4.80)	5.780	0.016	2.372 (1.154–4.875)

## Discussion

Periodontal diseases contribute to the development and progression of many other diseases (Nagpal et al., 2015). The importance of prevention, early diagnosis and treatment of periodontal diseases is thus far beyond the scope of dentistry.

Many factors, including oral hygiene, smoking, stress and anxiety, obesity and diabetes, contribute to the etiology of periodontal diseases (Slayton, 2006; Chaffee and Weston, 2010). Dental plaque is clearly an early step in the development of periodontal diseases, but many other factors are implicated in the progression of gingivitis to chronic periodontitis. Numerous studies have shown that susceptibility to periodontal diseases are affected by the polymorphisms of many genes, including interleukin-1 (*IL-1*) (Boukortt et al., 2015), tumor necrosis factor-α (*TNF-α*) (Heidari et al., 2013), transforming growth factor-beta (TGFβ) and 5-HTT (Ozer Yucel et al., 2015).

5-HTT is a critical part of 5-HT transmission. Many studies have implicated in the etiology of neuropsychiatric disorders (Kuzelova et al., 2010; Borkowska et al., 2015). Recent research also implicated 5-HT in the pathogenesis of autoimmune and chronic inflammatory diseases (Tanaka et al., 2014). Polymorphisms of the gene could alter the expression and function of (Hariri et al., 2002). Two polymorphic regions of the gene have been identified: a 44bp insertion-deletion in the promoter region (*LPR*) and a 17bp variable number of tandem repeat (VNTR) in the second intron (Lesch et al., 1994; Heils et al., 1996). The long (L) allele of LPR polymorphism increases 5-HT re-uptake (Greenberg et al., 1999), whereas the short (S) allele decreases the transcriptional efficiency of 5-HTT (Lesch et al., 1996). Multiple lines of evidence suggests that psychological factors also contribute in susceptibility to periodontal diseases (Goyal et al., 2013; Graetz et al., 2013; Reshma et al., 2013; Delgado-Angulo et al., 2015). A study by Costa et al. suggests that* t*he *5-HTT LPR* polymorphism is a risk of aggressive periodontitis in a Brazilian population (Costa et al., 2008). No studies have examined the potential association between polymorphisms and periodontal diseases in a Chinese population.

The current study confirms the association between *5-HTT* polymorphism and chronic periodontitis in the Chinese population. Specifically, the AC genotype, AC+CC genotype and C allele of the rs6354 polymorphism were more frequent in the subjects than in the healthy controls. Subgroup analysis suggested that women carrying the AC genotype of rs6354 have a higher risk of chronic periodontitis than women carrying other genotypes. A study by Su et al. indicated that rs6354 increases the risk of depressive symptoms (Su et al., 2009). Considering the notion that depression represents a risk factor of periodontal diseases (Hugo et al., 2006; Warren et al., 2014; Sundararajan et al., 2015), the association between rs6354 polymorphism and chronic periodontitis is reasonable. We did not find a significant association between the rs12449783 *5-HTT* polymorphism, but did show LD between rs6354 and rs12449783 and an increased risk of chronic periodontitis in subjects carrying the C-C haplotype C-C. The interaction between the two *5-HTT* SNPs requires further investigation.

The current study has several limitations. First, the sample size was relatively small for conducting subgroup analysis. Second, all study subjects were of Chinese Han ethnicity; whether the results could be extrapolated into other ethnic groups is unknown. Third, the results were adjusted only by age, sex and smoking status. Future studies with a larger sample size are needed to verify our findings.

In conclusion, *5-HTT* rs6354 but not rs12449783 polymorphism is associated with a susceptibility to chronic periodontitis in the Chinese Han population. The interaction between the two SNPs also seems to be associated with chronic periodontitis.

## Data Availability

The datasets for this manuscript are not publicly available because of relevant national biological and biomedical regulations. Requests to access the datasets with pure academic use should be directed to the corresponding authors.

## Ethics Statement

This study was reviewed and consented by the Ethics committee of Institute of Evidence-Based Medicine and Knowledge Translation, Henan University.

## Author Contributions

X-QR, X-TZ, and P-LG designed the study. LW and TD collected the data. X-QR and Y-YW examined data accuracy. C-YW and Y-YW performed the analysis. LW and X-TZ drafted the manuscript. P-LG reviewed the manuscript.

## Funding

This work was supported by the health commission of Hubei Province Scientific Reserch Project (WJ2019H035).

## Conflict of Interest Statement

The authors declare that the research was conducted in the absence of any commercial or financial relationships that could be construed as a potential conflict of interest.
